# Obesity in total hip arthroplasty—does it really matter? A meta-analysis

**DOI:** 10.3109/17453674.2011.588859

**Published:** 2011-09-02

**Authors:** Daniël Haverkamp, Mark N Klinkenbijl, Mathijs P Somford, G H Rob Albers, Harm M van der Vis

**Affiliations:** Department of Orthopaedic Surgery, Tergooi Ziekenhuizen, Hilversum, the Netherlands; Correspondence: Daniel@drhaverkamp.com

## Abstract

**Background and purpose:**

Discussion persists as to whether obesity negatively influences the outcome of hip arthroplasty. We performed a meta-analysis with the primary research question of whether obesity has a negative effect on short- and long-term outcome of total hip arthroplasty.

**Methods:**

We searched the literature and included studies comparing the outcome of hip arthroplasty in different weight groups. The methodology of the studies included was scored according to the Cochrane guidelines. We extracted and pooled the data. For continuous data, we calculated a weighted mean difference and for dichotomous variables we calculated a weighted odds ratio (OR). Heterogeneity was calculated using I_2_ statistics.

**Results:**

15 studies were eligible for data extraction. In obese patients, dislocation of the hip (OR = 0.54, 95% CI: 0.38–0.75) (10 studies, n = 8,634), aseptic loosening (OR = 0.64, CI: 0.43–0.96) (6 studies, n = 5,137), infection (OR = 0.3, CI: 0.19–0.49) (10 studies, n = 7,500), and venous thromboembolism (OR = 0.56, CI: 0.32–0.98) (7 studies, n = 3,716) occurred more often. Concerning septic loosening and intraoperative fractures, no statistically significant differences were found, possibly due to low power. Subjective outcome measurements did not allow pooling because of high heterogeneity (I_2_ = 68%).

**Interpretation:**

Obesity appears to have a negative influence on the outcome of total hip replacement.

Obesity has reached epidemic proportions in the USA, and the rest of the well-developed world is expected to follow. Since obesity is a well-documented risk factor for the development of osteoarthritis (Sturmer et al. 2000, Flugsrud et al. 2006), an increased need for joint arthroplasty in obese people can be expected. Surgery on obese patients can lead to longer duration of the operative procedures themselves, with higher complication rates and longer hospital stays, and some authors have even suggested refusal of elective surgery in obese patients (Fehring et al. 2007).

A controversy that has flared up during the last decennium is whether obesity might also influence the functional results and survival of total hip arthroplasty (THA), with studies showing either different or similar outcome compared to normal-weight patients. For both outcomes, different explanations have been postulated. McClung et al. (2000), for example, found that a higher BMI was associated with lower activity, resulting in less polyethylene wear in these patients, since wear is a function of use and not time. On the other hand, higher forces acting on the prosthesis in obese patients may lead to early loosening.

Generally, a person with a BMI between 25 and 30 is categorized as overweight, and someone with a BMI of greater than 30 is obese. In this meta-analysis, we evaluated the results of all published trials comparing outcome and survival of primary THA between different BMI groups (BMI of < 30 and of > 30). Our main research question was whether obesity has a negative effect on the short- and long-term outcome of total hip arthroplasty.

## Methods

Our search strategy was performed according to the recommendations of the Cochrane collaboration (Lefebvre et al. 2008). We searched the databases of Pubmed/Medline, the Cochrane Database of Systematic Reviews, and Embase from 1970 to 2010 regarding publications on obesity and THA. The search terms “arthroplasty”, “hip”, “weight”, “BMI”, and “obesity” were used. Furthermore, the lists of references of retrieved publications were manually checked for additional studies potentially meeting the inclusion criteria but not found by the electronic search. 2 investigators (DH and MK) independently reviewed the literature to identify relevant articles for full review. From the full text, using the above-mentioned criteria, the reviewers independently selected articles for inclusion in this review. Disagreement regarding the search was resolved by consensus, with arbitration by a third author (MS) when differences remained. Studies were included if they were comparative trials comparing the outcome of primary THA between different BMI groups. We included studies involving all types of cemented and non-cemented total hip prosthesis designs. Review articles, expert opinions, surgical techniques, and abstracts from scientific meetings were excluded. Only articles written in English were included. Studies were not blinded regarding author, affiliation, or source (Jadad et al. 1996). This systematic review and meta-analysis were done according to the PRISMA guidelines.

Our primary research question was to determine whether the outcome of primary THA is influenced by BMI. As short-term outcome, we selected the following complications: infection, hematoma, venous thromboembolism, and perioperative fractures. As medium- to long-term outcome parameters, we selected: dislocation, septic loosening, aseptic loosening, and subjective outcome at follow-up.

Methodology of the randomized clinical trials and controlled clinical trials was independently assessed by 2 reviewers (MK and MS) using the list of criteria recommended by the Cochrane Collaboration Back Review Group (van Tulder et al. 2003). Disagreement was resolved by group assessment. This frequently used list consists of 11 criteria for internal validity: 3 criteria related to selection bias, 4 criteria for performance bias, 2 criteria for attrition bias, and 2 for detection bias. Studies are considered to be of sufficient quality if at least 6 of the 11 validity criteria are met.

### Statistics

The data from the studies included were extracted by one reviewer (DH) using a pre-piloted data extraction tool, and they were verified by the second reviewer (MS). Then the available data from the selected studies were pooled using the Review Manager software from the Cochrane Collaboration. For outcome variables with a continuous nature, a weighted mean difference was calculated with 95% confidence interval (CI). For the dichotomous variables, a weighted odds ratio (OR) with 95% CI was calculated using Review Manager software.

For the studies where continuous variables were reported with a range, the SD was calculated using the method described by Walter and Yao (2007). The heterogeneity of the studies included was calculated using I^2^ statistics. This measurement describes the percentage of variation across studies that is due to heterogeneity rather than chance (Higgins et al. 2003). We also assessed heterogeneity by means of a chi-square analysis, whereby a p-value of < 0.1 was considered to be suggestive of statistical heterogeneity.

## Results

**Figure 1.**
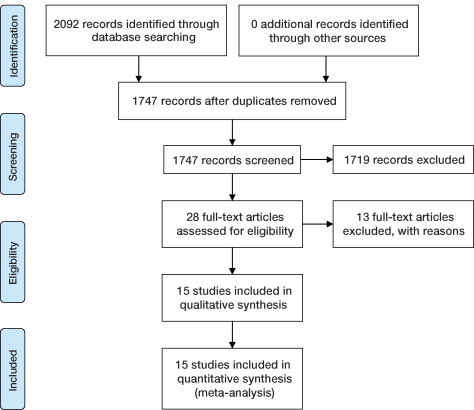
PRISMA flow diagram.

After consensus was reached, 15 studies were included for data analysis (Table 1 and Figure 1).

**Table 1.** The trials included

Author	Study type	Groups (BMI)	n	Follow-up	Outcome reported
Andrew et al. 2008	prospective	< 30, 30–40, > 40	1,421	mean 5 years	Survival, loosening, dislocation, complications, OHS
Bergschmidt et al. 2010	prospective	< 25, 25–30, 30–35	100	mean 2 years	HHS, WOMAC
Chee et al. 2010	prospective	< 30, > 30	110	mean 5 years	Survival, loosening, dislocation, complications
Dowsey et al. 2008	retrospective	< 25, 25–30, > 30	1,207	1 year	Infection
Ibrahim et al. 2005	retrospective	< 25, 30–40	459	1 year	Survival, loosening, dislocation, complications
Kessler et al. 2007	prospective	< 25, 25–30, > 30	67	3 months	Complications, WOMAC
Lehman et al. 1994	retrospective	< 30, 30–40, > 40	324	> 2 year	Survival, loosening, dislocation, complications
Lübbeke et al. 2007	prospective	< 30, > 30	2,636	> 5 years	Survival, loosening, dislocation, complications, HHS
McLaughlin et al. 2006	retrospective	< 30, > 30	285	> 10 years	Survival, loosening, dislocation, complications
Namba et al. 2005	retrospective	< 35, > 35	1,071	1 year	Loosening, dislocation, complications
Paterno et al. 1997	retrospective	< 30, > 30	380	> 2 years	Dislocation
Sadr Azodi et al. 2008	implant register	< 25, 25–30, > 30	2,106	mean 2 years	Dislocation
Søballe et al. 1987	retrospective	< 27, > 27	141	> 5 years	Survival, loosening, dislocation, complications
Yeung et al. 2010	retrospective	< 30, > 30	134	2–10 years	Survival, loosening, Harris hip score

**Figure 2.**
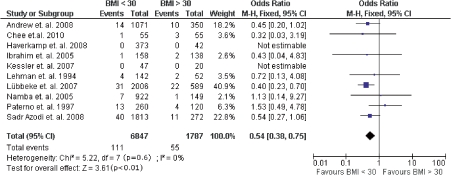
Forest plot, dislocations

Not all of the studies allowed retrieval of poolable data for the defined outcomes. Regarding dislocation, 10 studies (involving 8,634 patients) could be pooled and showed that dislocation occurred more often in patients with a BMI of > 30 (OR = 0.5, CI: 0.38–0.75). Heterogeneity was absent with an I^2^ of 0% (Figure 2). No subanalysis was performed for different types of prosthesis and approach, although all studies evaluated comparable approaches and implants.

**Figure 3.**
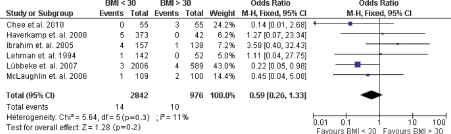
Forest plot, septic loosening.

**Figure 4.**
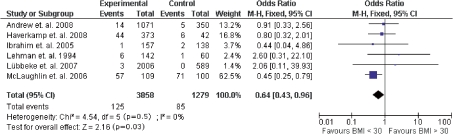
Forest plot, aseptic loosening.

Aseptic and septic loosening in the different groups were well documented in 6 of the studies. Septic loosening could be analyzed in 3,816 patients, which resulted in an OR of 0.6 (CI: 0.26–1.33), meaning that there was no statistically significant difference (Figure 3). For aseptic loosening, data from 5,137 patients could be pooled, and showed more aseptic loosening in patients with a BMI of > 30 (OR = 0.6, CI: 0.43–0.96); the forest plot is shown in Figure 4. Duration of the follow-up was not included in this analysis, but the amount of prosthesic loosening is certainly influenced by time. Since all studies included evaluated loosening in obese and non-obese patients over a similar follow-up period, the duration of the follow-up could be disregarded when pooling these events.

**Figure 5.**
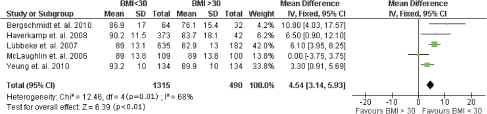
Forrest plot, Harris hip score.

Of the subjective outcomes, only the Harris hip score (HHS) was used often enough to allow pooling. Only follow-up periods of 2 years or more were pooled in this analysis, which showed a statistically significant mean difference of 5 (CI: 3.1–5.9) in 1,805 patients in 5 studies. Heterogeneity of these data was high, with an I^2^ of 68%, which did not allow pooling of the data. Furthermore, the minimal clinically important difference for the HHS is reported to be 4 points, which means that this difference was clinically relevant (Hoeksma et al. 2003) (Figure 5).

**Figure 6.**
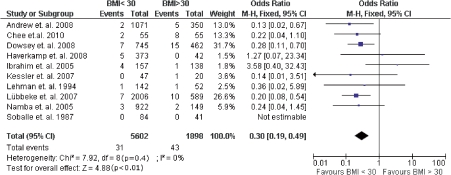
Forest plot, infection.

**Figure 7.**
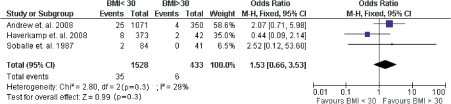
Forest plot, hematoma.

**Figure 8.**
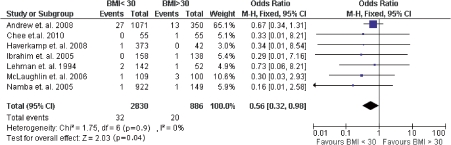
Forest plot, venous thromboembolism.

Of the early complications, infection was documented most consistently and precisely throughout the studies. 10 studies containing 7,500 patients could be pooled, giving an OR of 0.3 (CI: 0.19–0.49) and showing that infection occurred 3 times more often in obese individuals (Figure 6). Presence of a hematoma was not always mentioned or well-defined; thus, pooling was possible in only 3 studies with 1,961 patients, which did not reveal any statistically significant difference between the weight groups (OR = 1.5, CI: 0.66–3.5) (Figure 7). Venous thromboembolism (VTE) was often classified as deep vein thrombosis and pulmonary embolism. Since the underlying pathological mechanism is the same, we combined these numbers for pooling. Data from 3,716 patients in a total of 7 studies showed that VTE is more common in obese patients (OR = 0.6, CI: 0.32–0.98) (Figure 8).

**Figure 9.**
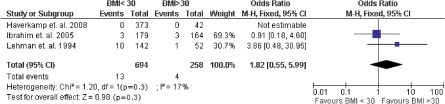
Forest plot, perioperative fracture.

Of the several intraoperative complications, fracture correlated best with difficulty of the procedure. However, pooling resulted in less than 1,000 patients in 3 studies, which in turn resulted in no statistically significant difference, possibly due to lack of power (Figure 9).

Of all the studies included, only 5 reported the preoperative co-morbidity, which gave a 2-fold higher co-morbidity in the obese (OR = 0.5, CI: 0.44–0.59) (5,747 patients, with an I^2^ of 55%). Correction for the presence of co-morbidity on the occurrence of complications is not possible in this meta-analysis.

Methodology in all the studies included scored less than 6 points, scoring all of them as low quality (Table 2). However, since all were comparable in quality, pooling of these studies was allowed.

**Table 2.** Methodology validity criteria per trial

Author	Selection bias	Performance bias	Attrition bias	Detection bias
Andrew et al. 2008	1/3	1/4	1/2	1/2
Bergschmidt et al. 2010	0/3	1/4	2/2	1/2
Chee et al. 2010	1/3	1/4	2/2	1/2
Dowsey et al. 2008	0/3	1/4	0/2	1/2
Haverkamp et al. 2008	1/3	1/4	2/2	1/2
Ibrahim et al. 2005	1/3	1/4	2/2	1/2
Kessler et al. 2007	0/3	1/4	2/2	1/2
Lehman et al. 1994	1/3	1/4	2/2	1/2
Lübbeke et al. 2007	1/3	1/4	2/2	1/2
McLaughlin et al. 2006	1/3	1/4	2/2	1/2
Namba et al. 2005	0/3	1/4	2/2	1/2
Paterno et al. 1997	0/3	1/4	2/2	0/2
Sadr Azodi et al. 2008	0/3	1/4	1/2	0/2
Søballe et al. 1987	1/3	1/4	2/2	1/2
Yeung et al. 2010	1/3	1/4	1/2	0/2

## Discussion

We found that the risk of performing THR on obese patients is certainly higher. Not only is the complication rate 3-fold higher; the longevity of the implant is also impaired. We therefore inform patients who are obese of these risks, and refer them to a multidisciplinary obesity outpatient clinic. If they do not lose weight, an advantage is that all co-morbidity of the patient is analyzed and his/her medical condition is optimized. We do not withhold THR from these patients, but we inform them of the risks associated with obesity regarding THR.

It is well known that obese people have more co-morbidity than people of normal weight. Theoretically, this co-morbidity could be the reason for the higher complication rates. It is not stated that obese people without co-morbidity have the same risk as obese people with co-morbidity, which of course is also true for the non-obese. However, if co-morbidity is the reason for higher complications and not the obesity itself, correction for the presence of co-morbidity should be performed on the data, especially since the studies that mention preoperative co-morbidity show a 2-fold higher incidence in the obese. Correcting for the presence of co-morbidity on the occurrence of complications was not possible in this meta-analysis.

The choice of cutoff point for BMI is based on a consensus that 30 is the borderline between obesity and non-obesity, but a BMI of 25 or more already means being overweight. Today, a BMI of between 25 and 30 is much more common and is beginning to be judged as more or less normal. To define the effect of weight on the outcome, it would be better to use the BMI as a continuous variable in the analysis. Not all studies used a BMI of 30 as the borderline; the oldest study of Søballe et al. (1987) used 27, a value that was popular in the 1980s. Namba et al. (2005) used a cutoff point of 35, and one study compared normal-weight patients (with a BMI of < 25) with obese patients (with a BMI of > 30), leaving out the patients with a BMI of between 25 and 30 (Ibrahim et al. 2005).

Previous studies have suggested that dislocation occurs more often in obese people (Paterno et al. 1997, Sadr et al. 2008). Correct placement of components may be more difficult in the obese. However, 2 studies have shown that component orientation is similar in obese patients and in those of normal weight (Pirard and De Lint 2007, Todkar 2008), but the power of these studies was probably not sufficient to answer this question. Another explanation for the higher dislocation rates in obese patients could be that surgery is more difficult and of longer duration, leading to more soft tissue damage and, subsequently, less intrinsic stability in the first few weeks and months.

We found a 3-times higher infection risk in obese patients. We noted, however, that the rates of septic loosening were not any different, which might indicate that only superficial infections occurred more often. Another explanation may be the lack of power, because of 24 events in approximately 4,000 patients. Another problem is that often the deep and superficial infections were not reported separately.

The difference between preoperative and postoperative Harris hip score may be more informative about the success of THR in the obese than simply HHS at follow-up, since it is not unlikely that obese patients score lower on subjective outcome measurements than normal-weight individuals, purely from the fact that they are obese and not as a reflection of the surgical result. In all studies reporting HHS, the only outcome measurement used frequently enough to consider pooling it was only reported as a follow-up value. Thus, the improvement in HHS could not be compared between groups. The HHS at follow-up had a heterogeneity that was too high to allow pooling. Apart from this, the measured difference in HHS between obese and normal-weight patients was barely higher than the minimal clinically important difference, which is 4 points for the HHS. This meta-analysis cannot therefore answer the question of whether subjective outcome differs between normal-weight and obese patients.
